# Temporal Progression of Excitotoxic Calcium Following Distal Middle Cerebral Artery Occlusion in Freely Moving Mice

**DOI:** 10.3389/fncel.2020.566789

**Published:** 2020-12-03

**Authors:** Ashley N. Nelson, Michael S. Calhoun, Ankur M. Thomas, Jennifer L. Tavares, Daniel M. Ferretti, Gregory M. Dillon, Yael Mandelblat-Cerf

**Affiliations:** Biogen, Cambridge, MA, United States

**Keywords:** dMCAO, calcium imaging *in vivo*, stroke, neuroprotection, MK-801, NMDA, excitotoxicity

## Abstract

Ischemic stroke is recognized as one of the leading causes of adult disability, morbidity, and death worldwide. Following stroke, acute neuronal excitotoxicity can lead to many deleterious consequences, one of which is the dysregulation of intracellular calcium ultimately culminating in cell death. However, to develop neuroprotective treatments that target neuronal excitotoxicity, it is essential to know the therapeutic time window for intervention following an ischemic event. To address this question, the current study aimed to characterize the magnitude and temporal progression of neuronal intracellular calcium observed following distal middle cerebral artery occlusion (dMCAO) in mice. Using the calcium fluorescence indicator, GCaMP, we tracked neuronal population response in freely moving animals immediately following dMCAO in both the core infarct and peri-infarct regions. Our results demonstrate that calcium excitotoxicity following artery occlusion can be generally characterized by two phases: a transient increase in activity that lasts tens of minutes, followed by a long, slow sustained increase in fluorescence signal. The first phase is primarily thought to represent neuronal hyperexcitability, defining our therapeutic window, while the second may represent gradual cell death. Importantly, we show that the level of intracellular calcium following artery occlusion correlated with the infarct size at 24 h demonstrating a direct connection between excitotoxicity and cell death in our stroke model. In addition, we show that administration of the NMDA antagonist MK-801 resulted in both a decrease in calcium signal and a subsequent reduction in the infarct size. Altogether, this study represents the first demonstration in freely moving animals characterizing the temporal progression of toxic calcium signaling following artery occlusion. In addition, these results define a critical time window for neuroprotective therapeutic intervention in mice.

## Introduction

Stroke is both the second leading cause of death globally (Guzik and Bushnell, 2017) and the number one cause of adult long-term disability in the United States. The World Health Organization estimates that 15 million strokes occur annually, leading to 5 million deaths and 5 million permanently disabled people. While advancements in preventative treatments, such as statins, antihypertensives, and ACE inhibitors, have improved risk management and contributed to the reduction in rates of stroke (Guzik and Bushnell, 2017), they have done little to abate the severity of subsequent impairments. The introduction of thrombolytic drugs, such as recombinant tissue plasminogen activator (rTPA), which can rapidly break down the fibrin in thrombi and reestablish cerebral blood flow (CBF), has markedly improved outcomes following stroke but is associated with a significant risk of hemorrhages and is also prone to re-occlusion (Moussaddy et al., 2018). More recently, advancements in stent and imaging technology have led to superior outcomes following mechanical thrombectomy (Moussaddy et al., 2018). Nevertheless, there remains great potential for the development of neuroprotective treatments which target pathological cellular processes initiated by stroke in order to further improve clinical outcomes.

Ischemic stroke occurs when arteries to the brain become narrowed or blocked, causing severely reduced blood flow. The resulting deprivation of key cellular metabolites such as oxygen, glucose, and ATP can trigger a number of detrimental cellular processes. Insufficient cellular ATP can lead to neuronal excitotoxicity through multiple mechanisms, including membrane depolarization following Na/K pump failure (Huang et al., 2015) and the inhibition of active glutamate reuptake from the synaptic cleft (Ketheeswaranathan et al., 2011; Kanyal, 2015). A majority of human stroke lesions are located in territories of the brain supplied by the middle cerebral artery (MCA) or one of its collaterals. Therefore, many labs have modeled cerebral ischemia preclinically through transient or permanent occlusion of the MCA, allowing the study of therapeutic intervention in these models (Llovera et al., 2014).

Evidence from both rodent and non-human primate studies indicates that following an ischemic event there are rapid increases in intracellular calcium levels (Murphy et al., 2008; Balbi et al., 2017). which are likely initially mediated through ionotropic NMDA receptors (Nishizawa, 2001; Wu and Tymianski, 2018). This overload of intracellular calcium can signal a wide range of pathological downstream cellular responses including the activation of calpains (Emerich et al., 2002) and the triggering of mitochondrial damage (Lesnefsky et al., 2017). Importantly, many of the signaling pathways downstream of excessive calcium culminate in cellular death, including necrosis or apoptosis (Orrenius et al., 2003). Together these data provide an indirect link between early neuronal excitotoxicity, calcium overload, and the cell death seen in ischemic stroke.

The discovery of neuronal excitotoxicity as a contributor to ischemic cell death led to a tremendous effort within the stroke research community to confirm whether blocking excessive glutamatergic transmission, or subsequent calcium influx, could reduce infarct volume and offer functional benefits to patients (Lai et al., 2014). While animal studies were mostly encouraging (Ozyurt et al., 1988; Park et al., 1988; Graham et al., 1993), clinical trials with either glutamate release blockers (Muir et al., 2000) or NMDA receptor blockers (Albers et al., 2001) failed to show efficacy. These failures can likely be attributed at least partially, to delays in drug administration that are inherent to clinical stroke trials with dosing occurring anywhere from 3 h (Albers et al., 2001) to as much as 9 h (Muir et al., 2000) following symptom onset (Lai et al., 2014). Recently, ambulance-based trials (Ankolekar et al., 2013) for ischemic stroke have begun and these trials will allow clinicians to test the hypothesis whether neuroprotection in stroke is possible with early intervention. In order to identify new targets suitable for neuroprotective treatments, it is essential to know the therapeutic time window for intervention following an ischemic event. To this end, *in vivo* imaging techniques, utilizing the calcium fluorescence indicator, GCaMP, can be used preclinically to probe intracellular calcium levels, and assess neuronal activity and/or ionic disturbances that may eventually lead to cell death (Russell, 2011). Previously many animal studies using calcium imaging required anesthesia during recording, which can have profound effects on neural activity, blood flow (Li et al., 2014), and consequently neurotoxicity (Harada et al., 1999). Therefore, in the current studies we employed *in vivo* fiber photometry to directly track the magnitude and temporal progression of neuronal intracellular calcium levels following distal middle cerebral artery occlusion (dMCAO) in freely moving mice.

Our results demonstrate that the significant increase in calcium following artery occlusion can be generally characterized by two phases: a transient increase in fluorescent signal that lasts tens of minutes, followed by a long slow increase in signal. We show that these two phases are present in both the infarct core and in the peri-infarct region and are significantly correlated with downstream cell death as measured by infarct volume 24 h after artery occlusion. Our data suggests that the first acute phase of calcium influx primarily represents neuronal hyperexcitability, as well as the window for neuroprotective intervention, while the second phase may represent gradual cell death. We show that administration of the NMDA antagonist MK-801 caused both a decrease in calcium signal and a subsequent reduction in the infarct size.

## Materials and Methods

### Animal Use

All experimental procedures using mice were approved by the Biogen Institutional Animal Care and Use Committee (IACUC). Male C57BL/6 mice were purchased from Jackson Laboratories (C57BL/6J, here referred to as JAX_*C*57_) and Charles River Laboratories (C57BL/6NCrl, here referred to as CRL_*C*57_). Mice were provided standard water and chow *ad libitum* throughout the duration of each experiment. Mice were acclimated at least 1 week prior to surgery, and all surgical procedures were conducted using aseptic technique as described in the Biogen IACUC Guidelines on Surgery in Rodents while wearing appropriate personal protective equipment (PPE).

### Fiber Photometry

#### Surgical Procedure

Mice were anesthetized using isoflurane in 100% oxygen (induction, 3%; maintenance, 1–2%) and placed into a stereotaxic apparatus (Kopf, Model 940 10 Small Animal Stereotaxic Instrument with Digital Display Console) on a temperature-controlled heating pad. Ophthalmic ointment was applied to the eyes. A 0.5 mm diameter burr hole was drilled over the mouse barrel cortex. To selectively record calcium induced fluorescence from pyramidal neurons in the projected infarct area, we injected 300 nL of AAV9-CAMKII-GCaMP6m (10^13 GC/ml, Vector Bio labs) into the barrel cortex using the following stereotaxic coordinates: (anterior-posterior for core area: 0.4 mm, for peri-infarct: −1.6 mm; dorsal-ventral, 0.7 mm; medial-lateral, 3 mm) of wildtype C57BL6 mice. Following the injection, an optic fiber (1 mm length, 400 μm diameter, NA = 0.48, metal ferrule, DORIC) was implanted 0.2 mm above injection area and secured to the skull with dental cement (Metabond dental cement. JAX_*C*57_ *N* = 23 core, CRL_*C*57_ *N* = 12 core, *N* = 8 peri-infarct). Additionally, a subset of mice had two implants to simultaneously record both from the core and the peri-infarct regions (CRL_*C*57_ *N* = 12, of which 6 were administered vehicle and MK-801).

#### Recordings

All experiments were conducted in an open arena in freely moving mice. Beginning 1 week post GCaMP injection, mice were habituated to the arena with fiber optic cable connected, and were administered 0.3 mL intraperitoneal (i.p.) injection of saline for habituation purposes.

*In vivo* fiber photometry was conducted as previously described (Chen et al., 2015). A fiber optic cable (“patch cord,” 1.5 m long, metal ferrule, 400 μm diameter; Doric Lenses) was firmly attached to the implanted fiber optic cannula with zirconia sleeves (Doric Lenses). Laser light (473 nm) was adjusted such that a light intensity of less than 0.05 mW entered the brain; emission light was passed through a dichroic mirror (Di02-R488-25x36, Semrock) and GFP emission filter (FF03-525/50-25, Semrock), before being collected by a sensitive photodetector (Newport part #2151). The signal was digitized at 1019 Hz using a TDT data acquisition software.

#### Recording Paradigm

Mice were recorded for a single day. On the recording day a patch cord was attached to the implanted optic fiber and the mouse was placed in an open arena. After 15 min, the mouse was injected i.p. with one of two treatments, 0.9% saline or 0.1 mg/kg MK-801, followed by an additional 15 min in the arena. Then, the mouse was disconnected from the recording setup and anesthetized before undergoing dMCAO surgery. After the dMCAO surgery, animal was maintained on a heating pad. Once the animal started to recover from anesthesia as indicated by response to toe pinch, it was reconnected to the patch cord and recorded for a minimum of an additional 3 h. Following the recording, the animal was returned to its holding cage.

### Drug Treatment

The open channel NMDA receptor antagonist (+)MK-801 hydrogen maleate was obtained from Sigma-Aldrich Corp. For all studies, MK-801 was dissolved in 0.9% saline for i.p. administration at 0.1 mg/kg at a time of 15 min prior to dMCAO surgery. This dose was found to significantly reduce baseline calcium responses in naïve animals (Supplementary Figure 2) while minimizing the occurrence of hyperlocomotion and stereotypy observed at higher doses.

### Distal Middle Cerebral Artery Occlusion (dMCAO) Surgery

Permanent occlusion of the distal middle cerebral artery (dMCAO) was performed as described in Doyle and Buckwalter (2014) Jax_*C*57_ (*N* = 21) or CRL_*C*57_ (*N* = 32) mice (14–16 weeks of age) were maintained under isoflurane anesthesia (1.5–2%) for the duration of the surgery. An incision was made between the orbit and auditory canal to expose and retract the temporalis muscle, exposing the skull superficial to the MCA. The MCA was exposed by drilling a 1–2 mm hole in the skull and then removing the meninges. The MCA was cauterized below the bifurcation using a low-temperature Bovie cauterizer. The temporalis muscle was reflected back into place, the incision was closed with surgical adhesive glue, and the animal was returned to the recording setup as mentioned previously. Sham mice Jax_*C*57_ (*N* = 2) and CRL_*C*57_ (*N* = 3) received the same surgery above, but with no cauterization of the MCA.

### TTC Staining

Mice were humanely euthanized in a CO_2_ chamber and immediately decapitated after last breath. Brains were removed and sectioned into 1 mm coronal sections using a brain mold. Brain sections were incubated for 10–15 min at room temperature in a 1.5% solution of 2,3,5-triphenyl tetrazolium chloride (TTC). Sections were then mounted onto slides and imaged. Infarct size in each section was quantified using ImageJ software (Doyle et al., 2012) by measuring the area of manually drawn regions of interest around the infarct (ROI1) and the entire ipsilateral hemisphere (ROI2). ROIs were drawn by an observer blinded to the animal groups. The percent infarct of ipsilateral hemisphere is calculated by (ROI1/ROI2) × 100. The average percent infarct of ipsilateral hemisphere is then calculated by taking the average of the percent infarct of ipsilateral hemisphere for all sections from a single animal.

### Data Analysis and Statistical Methods

Fiber photometry signal processing: Data analysis was performed in Matlab by custom written software. The signal was smoothed with a 1s squared bin window, and then down sampled to 1 Hz. The fractional change in fluorescence was computed according to the following equation: Δ*F*/*F* = (*F* – *F*0)/*F*0, where *F* is ongoing fluorescence measurement and *F*0 is the mean fluorescence in the 15 min prior to injection. Namely, we first calculated the average signal in the 15 min prior to injection (*F*0). Then, for each time point in the signal *F*(*t*), we subtracted the baseline average fluorescence (*F*0) and then divided by it. Δ*F*/*F* was calculated for each recording session (each mouse).

While the lengths of phase I and II are variable and defined by the signal dynamics, early and late signal are defined time windows: Early signal was defined as 0–30 min following dMCAO surgery, in accordance with the averaged length of phase I. Late signal was defined as the last hour of recording, 2–3 h post dMCAO surgery. Early and late signals were calculated as mean signal at time windows across the defined time windows.

Unless otherwise specified, all *p*-values were derived by Wilcoxon rank sum test for equal medians, with Bonferroni correction for multiple comparisons. Significance of correlation was calculated by testing the null-hypothesis, indicating that there was no relationship between the observed phenomena. All statistical tests and analyses were performed using Matlab (MathWorks).

## Results

### Experimental Paradigm for Fiber Photometry Recordings in Freely Moving Mice Immediately Following Permanent Distal Middle Cerebral Artery Occlusion (dMCAO)

**FIGURE 1:**
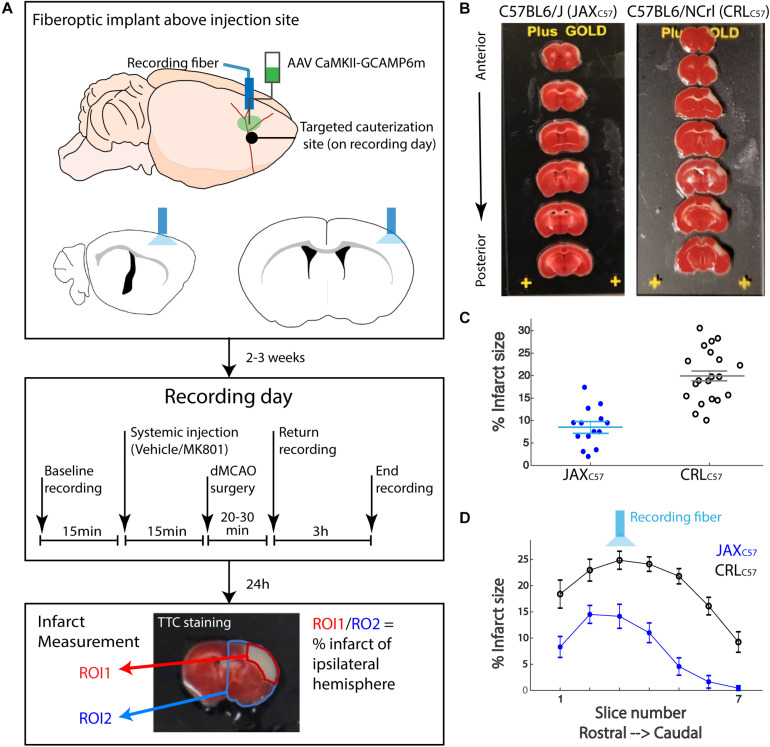
Experimental paradigm for fiber photometry recordings in freely moving mice immediately following permanent distal middle cerebral artery occlusion. **(A)** The experimental paradigm was divided into three stages. In the first stage **(upper panel)** mice received stereotactic injection of AAV9-CAMKII-GCaMP6m into the barrel cortex to achieve expression of the calcium reporter in the pyramidal neurons. The fiber optic implant was also placed directly above the injection site for photometry recordings. Following 2–3 weeks recovery, the second stage **(middle panel)** consisted of the recording day. Briefly, following a baseline photometry recording, mice are injected with either vehicle or MK-801 (0.1 mg/kg I.P.). 15 min later, mice are taken to a dMCAO surgery, then recordings continue for the next 3 h. 24 h later, in the third stage **(lower panel)**, TTC staining was performed on serial brain sections. Infarct size was calculated as a percentage of white infarct area (ROI1) divided by total tissue area (ROI2) on the ipsilateral hemisphere. **(B)** TTC staining on tissue sections from representative animals 24 h post dMCAO. Left-Jax_*C*57_; Right- CRL_*C*57_. White tissue reflects infarct. Top to bottom: 1 mm coronal sections, from most anterior to posterior. **(C)** Average infarct size across all brain sections for each mouse. Jax_*C*57_mice (blue asterisks, *N* = 14) show significantly lower infarct size as compared to CRL_*C*57_ mice (black circles, *N* = 20). *p* < 0.0001. Error bars represent SEM. **(D)** Averaged infarct size for each section across mice (Jax_*C*57_-blue; CRL_*C*57_ -black). Error bars denote SEM. Optic fiber targeted section 3.

The goal of the current study was to determine the role of intracellular calcium in cell death induced by an ischemic event. To this end we utilized GCAMP, a variant of the green fluorescent protein (cpGFP) linked to the calcium-binding protein calmodulin (CaM) and the CaM-interacting M13 peptide. Calcium dependent conformational changes cause increased fluorescent signal with calcium binding. These variants have previously been shown to have the ability to detect neuronal action potentials with high reliability (Chen et al., 2013). To record intracellular calcium in pyramidal neurons, we performed stereotaxic viral injections of GCAMP6m (AAV9-CaMKII-GCaMP6m, 300 nl, 10^13 GC/ml), and then implanted an optic fiber above the injection site (Figure 1A, top panel). The initial recording location was chosen based on calibration experiments in the distal middle cerebral artery occlusion model (dMCAO), demonstrating that this region of the cortex resides in the infarct core. Approximately 2–3 weeks later, each animal underwent a 30-min baseline recording session, followed by artery occlusion surgery, and then subjects were immediately returned to the recording chamber (Figure 1A, middle panel). The duration of anesthesia required for the surgery was kept at a minimum (20–30 min, see “Materials and Methods”), so as to not affect neuronal activity or cortical blood flow. The average time between artery occlusion and the start of post-stroke recordings was approximately 5 min. The total duration between baseline recordings and resuming to post-surgery recording, in which animals were removed from the recording setup and taken to surgery was ~30 min (29.65 ± 1.45; mean ± SEM). To quantify the volume of infarcted tissue following artery occlusion, animals were sacrificed 24 h after the recording session and brain sections were stained using 2,3,5-Triphenyltetrazolium chloride (TTC, Figure 1A, lower panel). Based on our previous results, the infarct was fully formed in the dMCAO model at the 24-h time point (Supplementary Figure 1). For sham surgeries, all steps of the procedure were followed, but artery cauterization was omitted. TTC staining confirmed that sham surgery did not result in any infarcted tissue (data not shown).

In an effort to increase our confidence that data from our dMCAO surgical model was robust and reproducible, we used C57Bl/6 mice from two different vendors, Jackson (referred as Jax_*C*57_) and Charles River Laboratories (referred as CRL_*C*57_). At 24 h following artery occlusion, the volume of infarcted tissue spanned up to 7 mm rostral to caudal and covered much of the somatosensory cortex, as depicted by the examples in Figure 1B (left panel – JAX_*C*57__;_ right panel – CRL_*C*57_). Interestingly, we found that overall CRL_*C*57_ mice had significantly larger infarcts (*n* = 20, 19.93 ± 1.313%, mean ± SEM), when compared to the Jax_*C*57_ sub-strain (*n* = 13, 9.099 ± 1.105%, *p* < 0.0001; Figure 1C). Investigating the infarcted tissue section by section, our data indicates that although CRL_*C*57_ mice had significantly larger infarcts across rostral to caudal sections, there was no interaction between mouse sub-strain and section position when analyzed by 2-way ANOVA (Figure 1D; Sub-strain factor *p* < 0.0001; section factor *p* < 0.0001; Interaction *p* = 0.2434). These results suggest that although there were overall differences in the size of infarcted tissue between mouse sub-strains, the infarct core (located in both groups at section 3) and pattern of tissue damage was similar between groups.

### Optical Imaging Reveals Two Distinct Temporal Phases of Increasing Intracellular Calcium Following dMCAO Surgery

**FIGURE 2:**
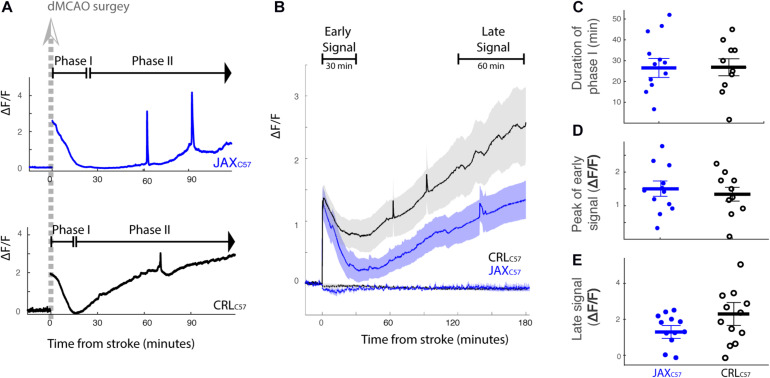
Fluorescence signal reveals two distinct temporal phases of intracellular calcium concentration following permanent dMCAO. **(A)** Fluorescent calcium signal recorded from a representative Jax_*C*57_ mouse (blue, upper trace) and CRL_*C*57_ mouse (black, lower trace) in response to dMCAO. At time zero, animal was disconnected from the recording and taken for dMACO surgery. Recording pre and post-surgery were concatenated in the data presentation. Fluorescent Signal is represented as the change in signal from baseline, Δ*F*/*F0*, where F0 is an average of the signal from the first 15 min of recording. **(B)** Averaged signal across mice (Jax_*C*57_ *N* = 12; CRL_*C*57_ *N* = 10). Shaded area denotes SEM. Dashed lines reflect response to sham surgery (Jax_*C*57_ *N* = 2; CRL_*C*57_ *N* = 3). The defined early and late time windows are denoted in black. **(C)** Distribution of the duration of phase I across mice. No significant difference between the mouse sub-strains (*p* > 0.2). **(D)** Peak response to dMCAO in early signal (measured by 95-percentile value in the time window 0–30 min). No significant difference between the mouse sub-strains (*p* > 0.2). **(E)** Late response to dMCAO. No significant difference between the mouse sub-strains (*p* = 0.09). For all panels, Jax_*C*57_ is depicted in blue, CRL_*C*57_ in black.

In the current experiments, fiber photometry was used to image intracellular calcium as a proxy of neuronal activity and/or ionic disturbances following an ischemic event. Our data demonstrates two distinct phases of increasing calcium signal in response to middle cerebral artery cauterization. The first phase peaked immediately after surgery and this transient increase lasted for ~26 min before returning to baseline levels. The second phase began immediately after the first phase and was represented by a gradual increase in signal out to the maximum recording time of 3 h post-surgery (examples from representative mice – Figure 2A). Figure 2B depicts the average response following dMCAO surgery for each mouse sub-strain (full lines; Black – CRL_*C*57_, Blue – Jax_*C*57_; shaded areas denote SEM). Notably, although our previous data demonstrates significant differences in the level of infarcted tissue between these groups, the time course was strikingly consistent between sub-strains. The duration of phase I, defined by the time point where the signal transitions from a return to baseline to slowly increasing, occurred at similar timepoints between groups (Figure 2C, average time post dMCAO, Jax_*C*57_ 26.6 min, CRL_*C*57_ 26.8 min). In order to quantify the calcium signal representative of each temporal phase, fluorescence signal was averaged across defined time windows (as depicted in Figure 2B; Early, 0–30 min; Late, 2–3 h following dMCAO surgery. See “Materials and Methods”). For the early time window, we noted that the peak response, as measured by the 95th percentile of the early signal, was similar between sub-strains (Figure 2D). Additionally, for both mice sub-strains the late signal was significantly higher than their early signal (Jax_*C*57_ *p* = 0.01, CRL_*C*57_ *p* = 0.025). However, although there was a trend indicating an increase in the late calcium signal for CRL_*C*57_ in comparison to Jax_*C*57_, this was not found to be significant (*p* = 0.09, Figure 2E).

Importantly, across all of our experiments, mice that underwent sham surgery (Figure 2B dotted lines) did not show any increase in calcium signal. If at all, they showed only a minimal and transient decrease in activity, suggesting also that the anesthesia did not have a prominent effect on the subsequent neural activity. This data represents the first characterization of the temporal progression of neuronal calcium levels in freely moving mice following dMCAO surgery.

### The Magnitude of Calcium Increase Following dMCAO Is Correlated With Infarct Size

**FIGURE 3:**
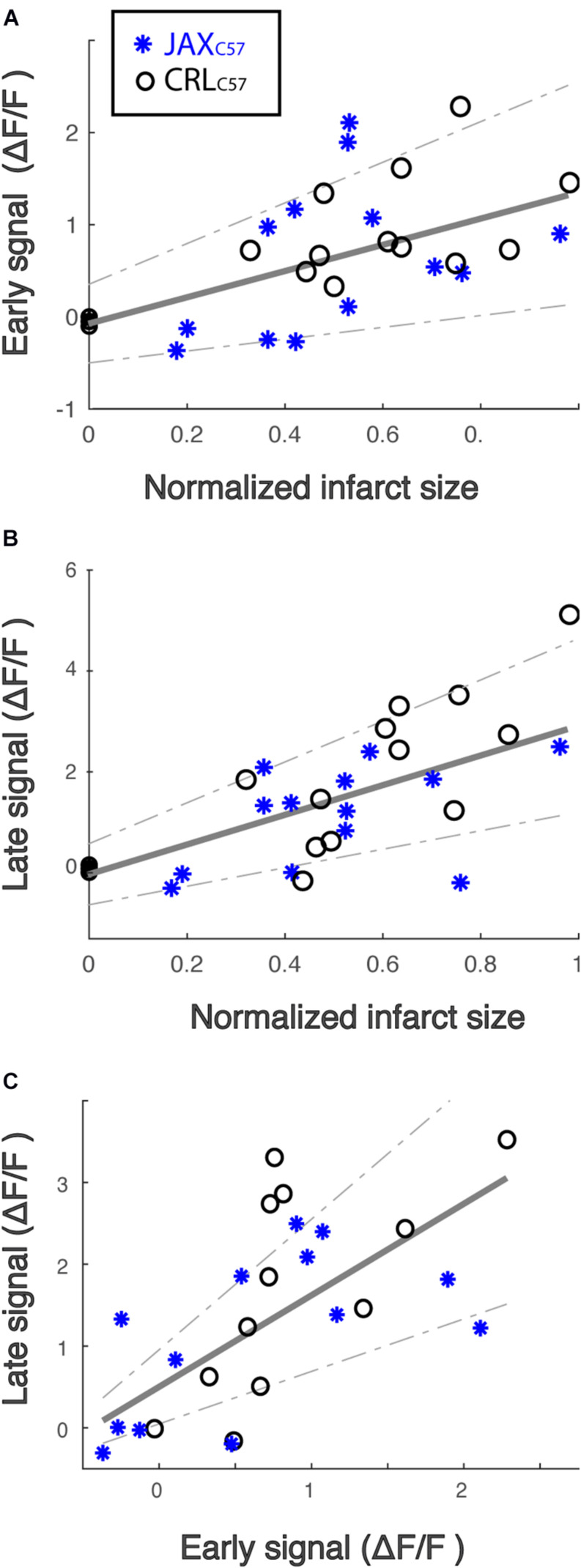
The magnitude of calcium increase following dMCAO is correlated with infarct size. **(A,B)** Normalized infarct size, calculated as the average infarct size across all sections for each mouse (*x*-axis), is correlated with both the mean early **(A)** and late **(B)** signal (*y*-axis). *R*^2^ = 0.24 and 0.39, respectively. Infarct sizes for each mouse was normalized by the maximal infarct for each sub-strain. Jax_*C*57_ Asterisks; CRL_*C*57_ circle. **(C)** The magnitude of late signal (*y*-axis) is correlated with early signal (*x*-axis). *R*^2^ = 0.48. For all panels, correlation fit for the combined data set of both mouse sub-strains and confidence interval are denoted in gray. For all panels, Jax_*C*57_ is depicted in blue, CRL_*C*57_ in black.

To determine potential relationships between the observed calcium signal increase and cell death, we inspected correlations between calcium signal at the early and late time windows and eventual total infarct size. Given the differences in the range of infarct sizes between mouse sub-strains, infarct size was normalized to the maximal infarct per sub-strain (see “Materials and Methods” for details). Our data demonstrates a significant correlation between final infarcted tissue volume and the magnitude of the calcium signal at the infarct core for both the early (Figure 3A; All animals, *p* = 0.005) and late time windows (Figure 3B; All animals *p* = 0.001). Notably, these correlations were also significant when looking specifically at each mouse sub-strain (Early signal Jax_*C*57_ *p* = 0.019, CRL_*C*57_ *p* = 0.022; Late signal Jax_*C*57_ *p* = 0.005, CRL_*C*57_ *p* = 0.002). Interestingly, we also noted a strong correlation within subjects between the early and late signal (Figure 3C; *p* = 0.0004). Together, these data indicate that following an ischemic event, the magnitude of initial calcium increase from baseline may be predictive of future cell death.

### Pre-treatment With the NMDA Antagonist MK-801 Significantly Lowers Subsequent Calcium Signal and Infarct Size in the dMCAO Model

**FIGURE 4:**
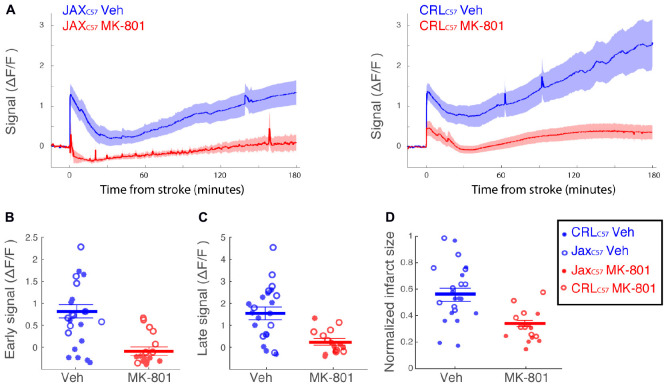
Treatment with the NMDA receptor antagonist, MK-801, significantly attenuates the calcium signal following dMCAO and decreases infarct volume. **(A)** Averaged response of fluorescent signal to dMCAO across mice for each sub-strain, following a vehicle (blue) or MK-801 (red) treatment. Shaded area denotes SEM. **(B,C)** Mean calcium signal following dMCAO is significantly reduced in response to pretreatment of MK801 in both early **(B)** and late **(C)** signal (*p* < 0.001). **(D)** Infarct size is significantly reduced in response to MK801 treatment (*p* < 0.001). **(B–D)** Lines for mean ± SEM for the combined set of mice are denoted. CRL_*C*57_ mice denoted in circles. Jax_*C*57_mice are denoted with asterisks. Vehicle – blue; MK-801 – red. CRL_*C*57_: *N* = 10 vehicle, *N* = 8 MK-801. Jax_*C*57_:*N* = 12 vehicle, *N* = 8 MK-801.

To examine if the observed calcium increase following dMCAO surgery was mediated by NMDA receptors, we treated animals with the NMDA antagonist MK-801 prior to artery occlusion. Our data shows that giving MK-801 (0.1 mg/kg i.p.) 15 min prior to dMCAO surgery significantly reduced the observed calcium increases as depicted by the averaged signal traces in both mouse sub-strains (Figure 4A). The observed decrease in calcium signal following MK-801 treatment was significant at both the early (Figure 4B; All mice *p* < 0.001) and late (Figure 4C; All mice *p* < 0.001) time windows (see Table 1A for signal values and significance per sub-strain). In order to determine whether this attenuation in calcium levels also reduced cell death downstream of ischemia, we measured infarct volume 24 h following artery occlusion. Our results show that MK-801 reduced infarct volume when compared to vehicle treated controls (Figure 4D and Table 1B; All mice *p* < 0.001). Together, this data indicates that the calcium response we see in freely moving animals following dMCAO is dependent on NMDA signaling. Importantly, lowering calcium influx with MK-801 was correlated with a significant decrease in cell death and infarct size (Supplementary Figure 3).

**TABLE 1:** Effect of MK-801 on calcium signal and infarct.

		Vehicle		MK-801		*p*-value Veh. vs. MK-801
		mean	s.e.m	mean	s.e.m	
**A**. Signal Averaged response to	Both_*C*57_ early	0.8	0.14	−0.04	0.09	0.001
dMCAO across early/late* time window	Both_*C*57_ late	1.61	0.27	0.22	0.12	0.0001
	Jax_*C*57_ early	0.63	0.23	−0.28	0.03	0.001
	Jax_*C*57_ late	1.14	0.28	0.05	0.16	0.002
	CRL_*C*57_ early	0.98	0.17	0.2	0.13	0.005
	CRL_*C*57_ late	2.12	0.43	0.38	0.18	0.007
**B**. Infarct Normalized measure (0 to 1)	Both_*C*_57	0.56	0.04	0.32	0.023	0.0001
across sub-strains of %infarct of	Jax_*C*_57	0.5	0.06	0.26	0.03	0.01
ipsilateral hemisphere	CRLC57	0.62	0.056	0.38	0.04	0.001

*Early/late time window: 0–30 min/2–3 h, post dMCAO, respectively.

### The Caudal Peri-Infarct Area Experiences Lower Calcium Load Following dMCAO

**FIGURE 5:**
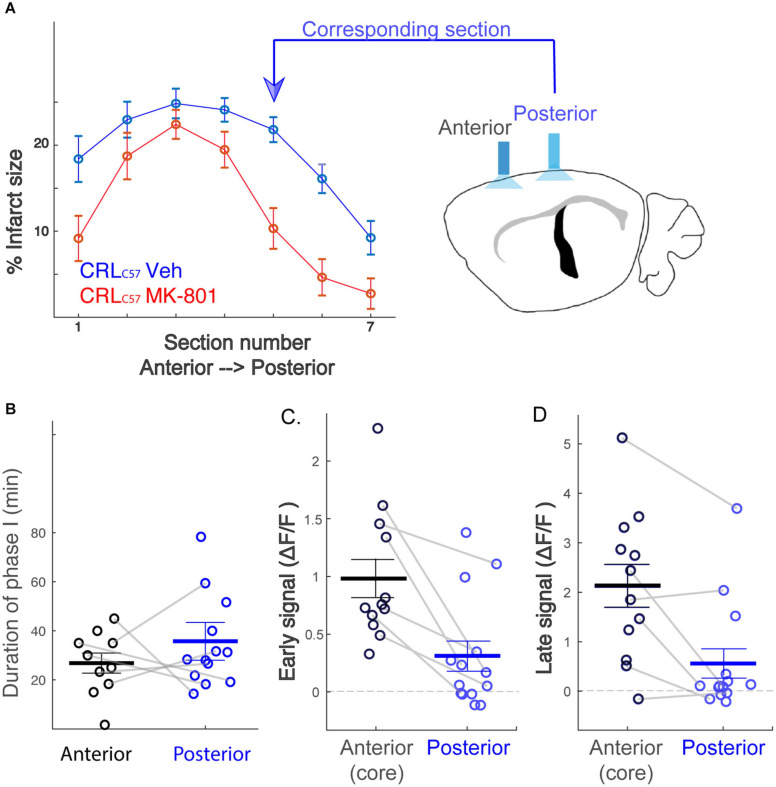
The posterior peri-infarct area experiences lower calcium load following dMCAO. **(A)** MK-801 reduces infarct size primarily at posterior sections (section 5,6; *p* < 0.01). Therefore, new “Posterior” recording site targeted to the location corresponding to sections 5–6. **(B)** Distribution of the duration of phase I across mice. No significant difference between the recording sites (Anterior – black, Posterior – blue), *p* = 0.20. **(C,D)** The posterior signal is significantly smaller than the anterior signal, both for early (**C**, *p* = 0.003) and late (**D**, *p* = 0.008) time windows. Gray connecting lines represent mice for which anterior and posterior signals were recorded simultaneously.

**FIGURE 6:**
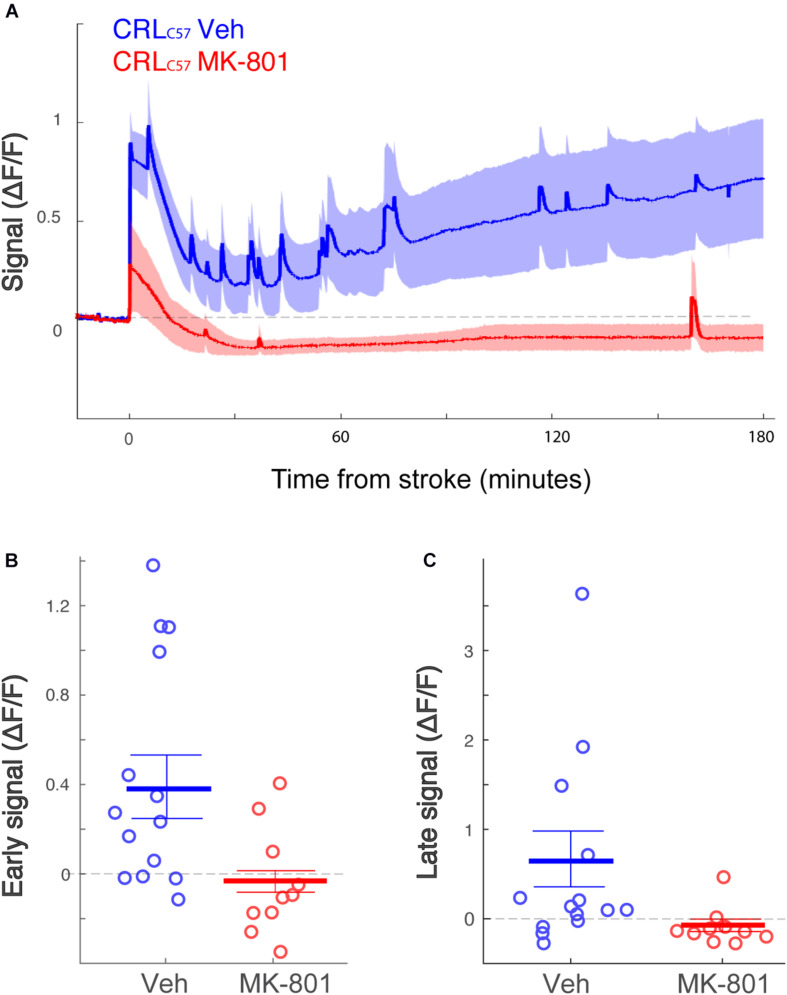
The posterior peri-infarct area more neuroprotection by MK-801 treatment. **(A)** Averaged response of fluorescent signal to dMCAO across CRL_*C*57_ mice following a vehicle (blue) or MK-801 (red) treatment. Shaded area denotes SEM. **(B,C)** Mean calcium signal following dMCAO is significantly reduced in response to pretreatment of MK801 in both early (**B**, *p* = 0.024) and late (**C**, *p* = 0.008) time windows and becomes insignificantly different from zero. For all panels: Vehicle – blue; MK-801 – red. CRL_*C*57_ Anterior recordings: *N* = 12 vehicle; *N* = 10 MK-801. Posterior recordings *N* = 14 vehicle, *N* = 10 MK-801.

To expand on our previous dMCAO results, we investigated whether MK-801 offered similar neuroprotection across the entire volume of infarcted tissue, rostral to caudal, in CRL_*C*57_ mice. Based on serial sections, we demonstrate a significant effect of MK-801 when compared to vehicle treated controls in the posterior sections (Figure 5A). This indicates that in our dMCAO model, we have a reproducible area representing both the infarct core (maximal infarct, unsalvageable – sections 2–3), as well as salvageable penumbral tissue 2–3 mm posterior to that region, which we have referred to as the peri-infarct. To determine whether differences in calcium signal may underlie the different vulnerabilities of these brain regions to cell death, we carried out additional experiments moving the recording fiber 2 mm posterior from the original location (Figure 5A; coordinates relative to Bregma: 3 mm lateral, 1.6 mm posterior. Corresponding to section #5 in Figure 1B, CRL). Similar to the anterior recording area, fiber photometry recordings from vehicle treated animals at this location also revealed a similar biphasic calcium response following artery cauterization, and the duration of Phase I was not significantly different between anterior and posterior recording locations, even though these fibers were separated by 2–3 mm (Figure 5B, *p* = 0.20). Interestingly, the magnitude of the calcium signal at the posterior location was significantly smaller when compared to recordings from the infarct core for both the early (Figure 5C, *p* = 0.003) and late signal (Figure 5D, *p* = 0.008). Together, this data suggests that reduced initial calcium influx within the peri-infarct area may make this region salvageable for future neuroprotection by MK-801. To test this, we set out to inspect the effect of MK-801 administration on the calcium signal recorded from the posterior location. Averaged response across mice (Figure 6A) reveals that MK-801 caused a substantial decrease in calcium signal (red) as compared to vehicle administration (blue). MK-801 significantly reduced the calcium signal in the posterior peri-infarct area during both early (Figure 6B *p* = 0.011) and late (Figure 6C, *p* = 0.013) time windows. Notably, MK-801 decreased the late signal significantly more than it did in the infarct core (*p* = 0.045). In fact, the late signal was decreased to zero, coupled with the lower infarct.

## Discussion

Currently, the only therapeutic options following an ischemic event focus on restoring blood flow to hypoxic brain areas. To complement reperfusion therapy, neuroprotectants could be utilized to slow down the spread of the ischemic core, preserve at risk tissue, and potentially increase the number of patients with successful outcomes following thrombolysis or thrombectomy (Savitz et al., 2017). In order to identify new drug targets for neuroprotection, we sought to characterize the temporal and spatial progression of calcium induced excitotoxicity and cell death by directly monitoring *in vivo* intracellular calcium in the mouse dMCAO model. Our results show a clear biphasic calcium response following artery occlusion with an extremely consistent timescale and tissue distribution between subjects and mouse sub-strains. In addition, our results demonstrate a clear correlation between the levels of intracellular calcium and the subsequent volume of infarcted tissue. Overall, these results establish both a time window to test neuroprotectants in the dMCAO model and suggest that targeting the influx of calcium following ischemia or blocking cell-death substrates activated by calcium overload represent promising neuroprotective targets in ischemic stroke.

In order to obtain a clear understanding of how ischemia affects the magnitude and temporal progression of neuronal excitability, avoiding the use of anesthesia during recording is critical. In humans, it is widely accepted that anesthetics affect consciousness through modulation of neural activity. Additionally, anesthetics like isoflurane, a potent vasodilator, have been shown to directly affect the volume of infarcted tissue in rodent models of stroke (Sakai et al., 2007; Seto et al., 2014) by increasing cortical blood flow to peri-infarct area (Iida et al., 1998) or by altering GABA and glutamatergic transmission (de Sousa et al., 2000; Kotani and Akaike, 2013). Therefore, in our studies intracellular calcium, neuronal hyperexcitability and infarct area may be directly impacted by using anesthesia. To minimize this, all recordings were carried out in awake, freely moving mice and the duration of anesthesia during artery occlusion surgery was kept consistent and at a minimum between animals (average anesthesia time for surgery was 15 min). The current study is the first to track intracellular calcium by fiber photometry in awake animals following artery occlusion, and this allowed a more accurate characterization of neuronal population responses over time.

Our *in vivo* recordings show that the calcium signal post-dMCAO surgery was characterized by two phases – a transient increase in activity which returned to baseline after approximately 30 min, followed by a gradual increase that continued for at least 3 h. Importantly, although we show differences in both the magnitude of calcium response and vulnerable tissue area between two mouse sub-strains (CRL_*C*57_ and Jax_*C*57_), the time course of these calcium phases was similar. It was previously reported using *ex vivo* preparations of mouse spinal cord neurons (Tymianski et al., 1993b) or rat hippocampal neurons (Calvo et al., 2015), that exposure to excitotoxic glutamate caused a similar biphasic calcium response with an initial, transient phase that decays within minutes followed by a more sustained, secondary phase. In the current study, we believe that the first phase of calcium influx most likely reflects acute neuronal hyperexcitability as a result of ATP deprivation, while the second phase could represent a process previously termed delayed calcium deregulation (DCD; Tymianski et al., 1993a). DCD is considered a hallmark of glutamate excitotoxicity (Budd and Nicholls, 1996) and may be triggered by impaired mitochondrial bioenergetics (Abramov and Duchen, 2010) or by the activation of calpains (Araujo et al., 2010) leading to neuronal cell death. This hypothesis matches the time course of calcium signaling and infarct progression in our model, in which we clearly see expansion in the infarcted tissue between 2 and 6 h following artery occlusion (Supplementary Figure 1) coinciding with the gradual increase in calcium during the second phase. Importantly, our data demonstrates a strong correlation across subjects between the magnitude of calcium signal recorded and subsequent infarct volume 24 h after occlusion. This correlation was consistent for both time windows examined (early, 0–30 min vs. late, 2–3 h). Interestingly, we show a reduction in the magnitude of calcium signal measured in the peri-infarct region when compared to the infarct core, and because the peri-infarct region is also the area where we demonstrate salvageable brain tissue, this further reinforces the causal link between intracellular calcium load and cell death in our model. Together these results provide a defined therapeutic window to test neuroprotectant targets on either acute hyperexcitability or DCD with future opportunities to determine how each of these calcium phases affect infarct progression.

Our data supports the hypothesis that phase I reflects calcium-induced hyperexcitability. In the current work, mice demonstrated more than 2-fold increase in their calcium signal at the infarct core immediately after artery occlusion surgery. Interestingly, this first phase of calcium influx was close to its peak at the start of the recording session (approximately 5 min from surgery to recording), and this time frame was consistent when recording from different brain regions. Previous experiments have also noted large increases in calcium signals occurring 1–3 min following an ischemic event using a range of imaging tools including calcium indicator dyes (Murphy et al., 2008), fMRI probes (Savic et al., 2019), and genetically engineered GCaMP3 mice (Balbi et al., 2017). To determine the source of this first phase of calcium we administered MK-801, a known NMDA channel blocker (Foster et al., 1987), that has previously been shown to exert neuroprotective properties in multiple animal models (Ozyurt et al., 1988; Park et al., 1988). When administered prior to artery occlusion, MK-801 significantly reduced both the early and late time windows of calcium influx. Importantly, MK-801 also reduced the total infarct size 24 h after artery occlusion demonstrating an additional correlation between the levels of intracellular calcium and the downstream activation of cell death. We believe that the effects of MK-801 on infarct volume are mostly attributable to its effect in lowering the early influx of calcium following artery occlusion. This conclusion is supported by several pieces of data: first, when examining the calcium signal in sham mice, MK-801 at 0.1 mg/kg was able to lower baseline calcium fluorescence for a window of approximately 2 h (Supplementary Figure 2A), indicating that after this time we did not detect a pharmacodynamic effect of the drug at this dose. Additionally, we show that 3 h following dosing of MK-801 at 0.1 mg/kg, the concentration of free drug in the brain is below the projected IC_50_ for this compound (63 nM; Supplementary Figure 2B). This validates that the effect of MK-801 on the late calcium signal (2–3 h after occlusion, equivalent to approximately 3–4 h after dosing) was not due to direct target engagement of NMDA receptors. Finally, our data shows a significant correlation within subjects between the early and late calcium signals, suggesting that the later phase, which we hypothesize is the result of cell death, may be dependent on the magnitude of the early calcium signal. To fully test this hypothesis, we would need to administer MK-801 after Phase I and determine if the reduction in infarct volume we observed is lost. Altogether, our findings suggest that early NMDA receptor-mediated calcium influx contributes to later cell death and infarct progression, and therefore early neuronal hyperexcitability may be the initial trigger which ultimately culminates in neuronal cell death.

Spreading depolarizations are recognized as an acute hallmark of stroke (Lauritzen et al., 2011; Dreier and Reiffurth, 2015), and this phenomenon is both highly evolutionarily conserved (Spong et al., 2016) and found in the clinical setting (Strong et al., 2002). Importantly, in addition to excitotoxicity occurring at the single cell level, waves of depolarization could be responsible for propagating glutamatergic and ionic imbalances across the tissue, impacting infarct progression into the penumbra (Lauritzen et al., 2011). In the current study, our setup does not allow for us to determine the contribution of these waves to overall calcium signal, but we do not believe that they have a significant contribution to the observed signal. Our results indicate an identical time window for the first phase of calcium in both the anterior and posterior recording locations, even though these fibers were separated by 2 mm. If spreading depolarizations made up a significant proportion of the observed calcium signal, then we would have expected a delay in the initial calcium peak, or in the return to baseline, when recording from the peri-infarct region compared to the infarct core.

In ischemic stroke it is likely that calcium dysregulation occurs across different cell types and at both the single cell and tissue level. In the current study we aimed to track pyramidal neurons using an AAV with the CAMKII promoter to drive GCaMP expression in these neurons. However, it is well known that cerebral ischemia can also elicit robust neuroinflammatory responses through the activation of non-neuronal cell types in the brain including astrocytes and microglia (Hersh and Yang, 2018). Previously it has been shown that under conditions of ischemia or cell damage, both astrocytes and microglia respond with very fast intracellular, calcium spikes (Chuquet et al., 2007; Eichhoff et al., 2011); however, the role this activation plays in the progression of ischemic cell death seem to be opposed depending on cell type. For example, IP3 receptor knockout mice show a reduction in astrocytic calcium transients following permanent MCAO surgery and consequently a reduction in infarct size (Rakers and Petzold, 2017), suggesting a connection between pathological astrocytic excitability and downstream cell death. In contrast, elimination of microglia from the brain results in a dramatic increase in infarct size (Szalay et al., 2016), demonstrating that activation of microglia following ischemia is critical in the brain’s response to injury. Future studies are necessary to determine how the time frame of astrocyte and microglia calcium responses match up with our neuronal data and to determine if the block of neuronal calcium with agents like MK-801 would subsequently alter astrocytic responses.

In the preclinical assessment of neuroprotectant targets, the reproducibility of *in vivo* experimental results can be affected by a wide range of variables including the age, gender, or strain of mouse tested. In the current study, we have surprisingly shown that differences in infarct size can occur even within the same C57BL/6 mouse strain, with CRL_*C*57_ mice exhibiting significantly larger infarct volumes when compared to Jax_*C*57_. Interestingly, this is not the first observed phenotypic difference between C57BL/6 sub-strains (Bryant et al., 2019), and while determining the underlying cause of the observed difference is outside the scope of this paper, multiple possibilities exist. First, genetic differences between C57BL/6 mouse sub-strains have previously been reported (Specht and Schoepfer, 2001; Mekada et al., 2009), and it is possible that single nucleotide polymorphisms (SNPs) on specific genes could confer protection or increase susceptibility following ischemia. In addition, it has been shown that different strains of mice have differences in the architecture of their brain vasculature which ultimately affects infarct size following MCA occlusion (Kang et al., 2015). Therefore, it is also possible that the C57BL/6 sub-strains may differ anatomically as well. Another potential difference between the Jax_*C*57_ and CRL_*C*57_ mouse sub-strains used in this paper may be differences in gut microbiome composition. Interestingly, Jackson Laboratory has reported that the gut microbiome can differ between mice of the same sub-strain purchased from different vendors (Low-Marchelli, 2019). Gut microbiota dysbiosis has been shown to impact stroke lesion size and outcome in animal models (Benakis et al., 2016; Singh et al., 2016), and since the mouse sub-strains used here were purchased from separate vendors, infarct size variation may be due to differences in microbiome composition. Intriguingly, our results show that CRL mice trended toward having a larger late calcium signal in comparison with the JAX substrain. This trend may provide further evidence that the late calcium signal in our model is a correlate of neuronal death. Because these sub strains did not show a difference in the magnitude of early calcium signal it is interesting to speculate that strain specific vasculature or genetic differences could be underlying this effect on final lesion size. Overall, more research is needed to determine why we observed sub-strain differences in infarct size post-dMCAO; however, it is telling that even with all of these potential differences, the biphasic calcium response we describe here following ischemia is consistent.

The dMCAO model used in the present study results in the permanent loss of blood flow to the brain regions supplied by the MCA. This model provides multiple benefits when compared to traditional transient stroke models including lower subject mortality, lower variability in infarct size and location, and a shorter surgical duration, allowing for less anesthesia exposure (Doyle et al., 2012; Llovera et al., 2014). Therefore, this model allowed us to quickly assess neuronal calcium responses to ischemia in a setting with low variability. Unfortunately, a limitation of a permanent artery occlusion model is the lack of face translatability to the clinical settings of ischemic stroke. Currently, the only clinically proven treatment for stroke is to re-establish blood flow in the artery, either chemically through rTPA or mechanically by thrombectomy (Moussaddy et al., 2018). For this reason, repeating these studies using a transient stroke model, which allows for either pharmacological or mechanical reperfusion of the brain tissue following the occlusion, would allow for increased translatability of our findings.

Although previous neuroprotectant trials in acute ischemic stroke have failed, we still believe there is potential with this strategy. Stroke therapy has entered an era of highly effective reperfusion therapies (Goyal et al., 2016), and current clinical trials are knocking down previous hurdles around acute ambulatory care (Saver et al., 2015; Wendt et al., 2015). In the ENACT phase 2 trial of patients receiving surgical aneurysm repair (ClinicalTrials.gov NCT00728182), pre-treatment with NA-1 (Tat-NR2B9c; an inhibitor of postsynaptic density-95 protein binding to the NMDA2B subunit) was shown to be effective in reducing the number of ischemic infarcts when compared to placebo treated controls (Hill et al., 2012). NA-1 is also currently being tested in two separate Phase 3 trials (ClinicalTrials.gov: ESCAPE-NCT02930018; FRONTIER-NCT02315443). Though much further investigation is necessary, ESCAPE secondary findings showed that in a subgroup of patients who did not receive tPA, the potential for improving functional outcome, reducing infarct volume, and reducing mortality? (Hill et al., 2020). These trials are all clinical demonstrations that neuroprotection in acute ischemic stroke might be possible. In the development of new neuroprotective agents, understanding the window of tissue vulnerability for potential therapeutic intervention is extremely important. In the current study, by using fiber photometry in freely moving animals, we are able to describe the temporal and spatial progression of processes downstream of hypoxia without the confounds of anesthesia. In addition, our results demonstrate a clear correlation between the levels of intracellular calcium and the subsequent volume of infarcted tissue. In summary, the data provided here give insight into the relative timing of events associated with focal ischemia and can hopefully guide future therapeutic strategies targeting the mechanisms of cell death following stroke.

## Data Availability Statement

The raw data supporting the conclusions of this article will be made available by the authors, without undue reservation.

## Ethics Statement

The animal study was reviewed and approved by the Biogen Institutional Animal Care and Use Committee.

## Author Contributions

AN and MC established the DMCAO surgery and TTC analysis. JT and DF performed the stereotaxic surgeries. YM-C performed the recordings and data analysis. AN, MC, AT, GD, and YM-C contributed to the development of this research and writing the manuscript. All authors contributed to the article and approved the submitted version.

## Conflict of Interest

At the time that this work was performed, all authors were employees of Biogen.

## Supplementary Material

Supplementary Figure 1 Infarct is already formed at 6 h post-dMCAO. Average percent infarct size (ROI1/ROI2), measured by TTC staining is shown for several timepoints following dMCAO. Error bars denote SEM. No significant difference in infarct size was observed across the time points.

Supplementary Figure 2 MK-801 IP injection calibration. **(A)** Calcium fluorescent signal in response to different dosages of MK801 (depicted on the right). Signal showed significant decrease in activity for all dosages except for the lowest one (0.01 mg/kg). **(B)** Brain concentration of MK-801 at different time points after IP injection of 0.1 mg/kg MK-801 shows a significant decrease between time points (ANOVA test, *p* < 0.01).

Supplementary Figure 3 Treatment with MK-801 significantly attenuates both the calcium signal and infarct volume following dMCAO. **(A,B)** Responses to pretreatment of MK-801 result both in a decrease in infarct size and mean early **(A)** and late **(B)** signal. Jax_*C*57_Asterisks; CRL_*C*57_ circles. Jax_*C*57_Asterisks; CRL_*C*57_ circles.
