# Supplementary figures and images for: Temporal Progression of Excitotoxic Calcium Following Distal Middle Cerebral Artery Occlusion in Freely Moving Mice
Source: Front Cell Neurosci. 2020 Dec 3;14:566789. doi: 10.3389/fncel.2020.566789 (PMC7794019; doi:10.3389/fncel.2020.566789)

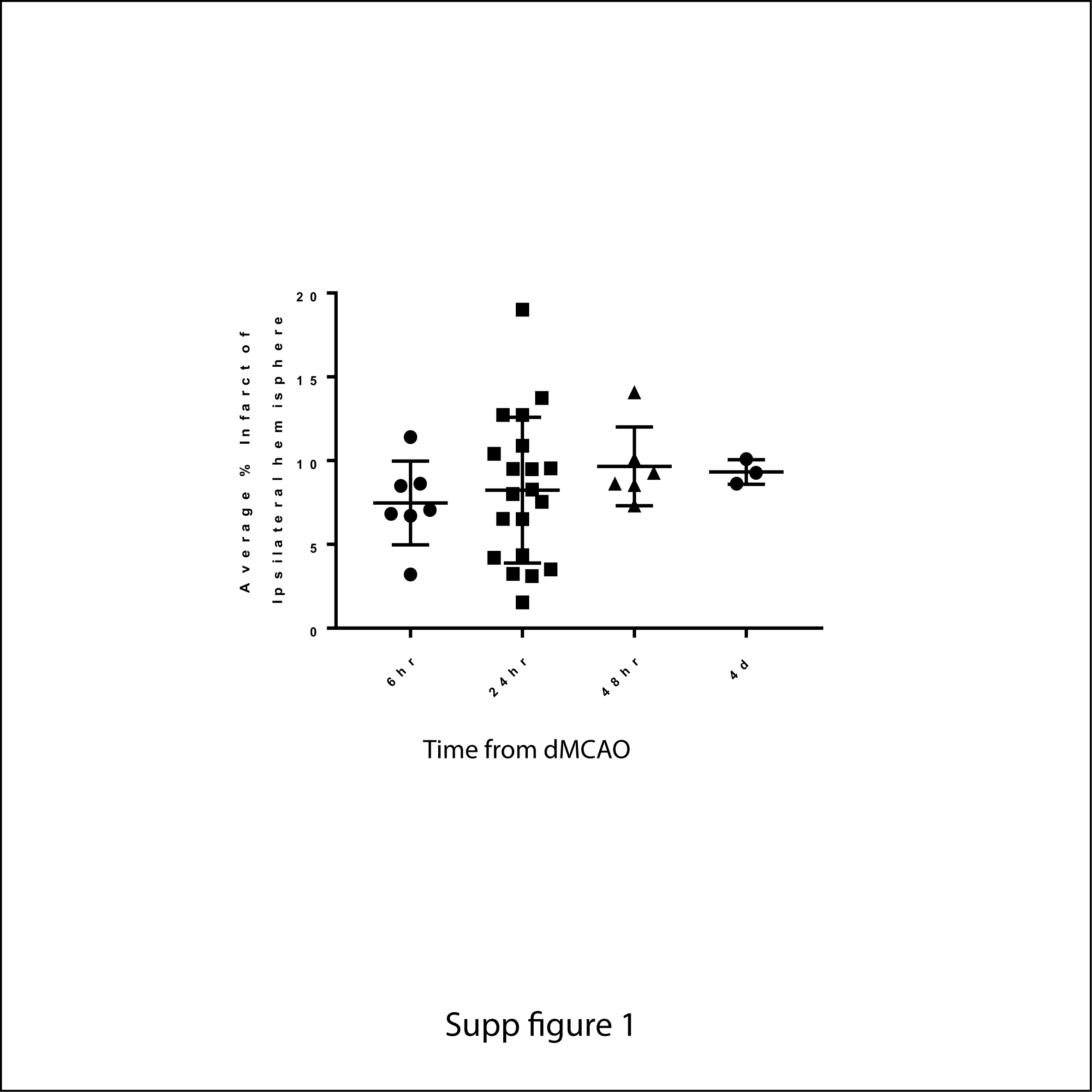

Supplement: Supplementary Figure 1 — Infarct is already formed at 6 h post-dMCAO. Average percent infarct size (ROI1/ROI2), measured by TTC staining is shown for several timepoints following dMCAO. Error bars denote SEM. No significant difference in infarct size was observed across the time points. [file Image_1.TIF]

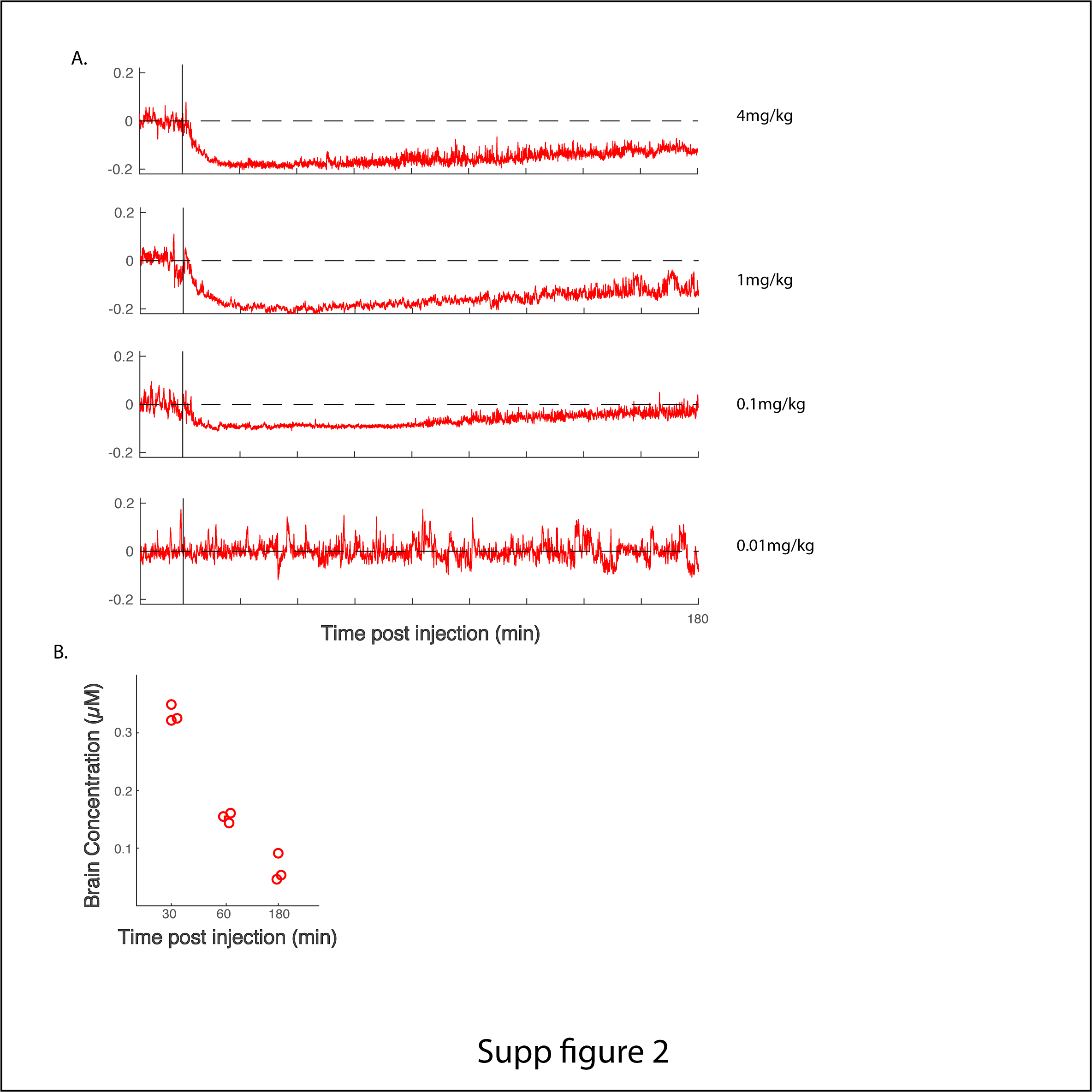

Supplement: Supplementary Figure 2 — MK-801 IP injection calibration. (A) Calcium fluorescent signal in response to different dosages of MK801 (depicted on the right). Signal showed significant decrease in activity for all dosages except for the lowest one (0.01 mg/kg). (B) Brain concentration of MK-801 at different time points after IP injection of 0.1 mg/kg MK-801 shows a significant decrease between time points (ANOVA test, p < 0.01). [file Image_2.TIF]

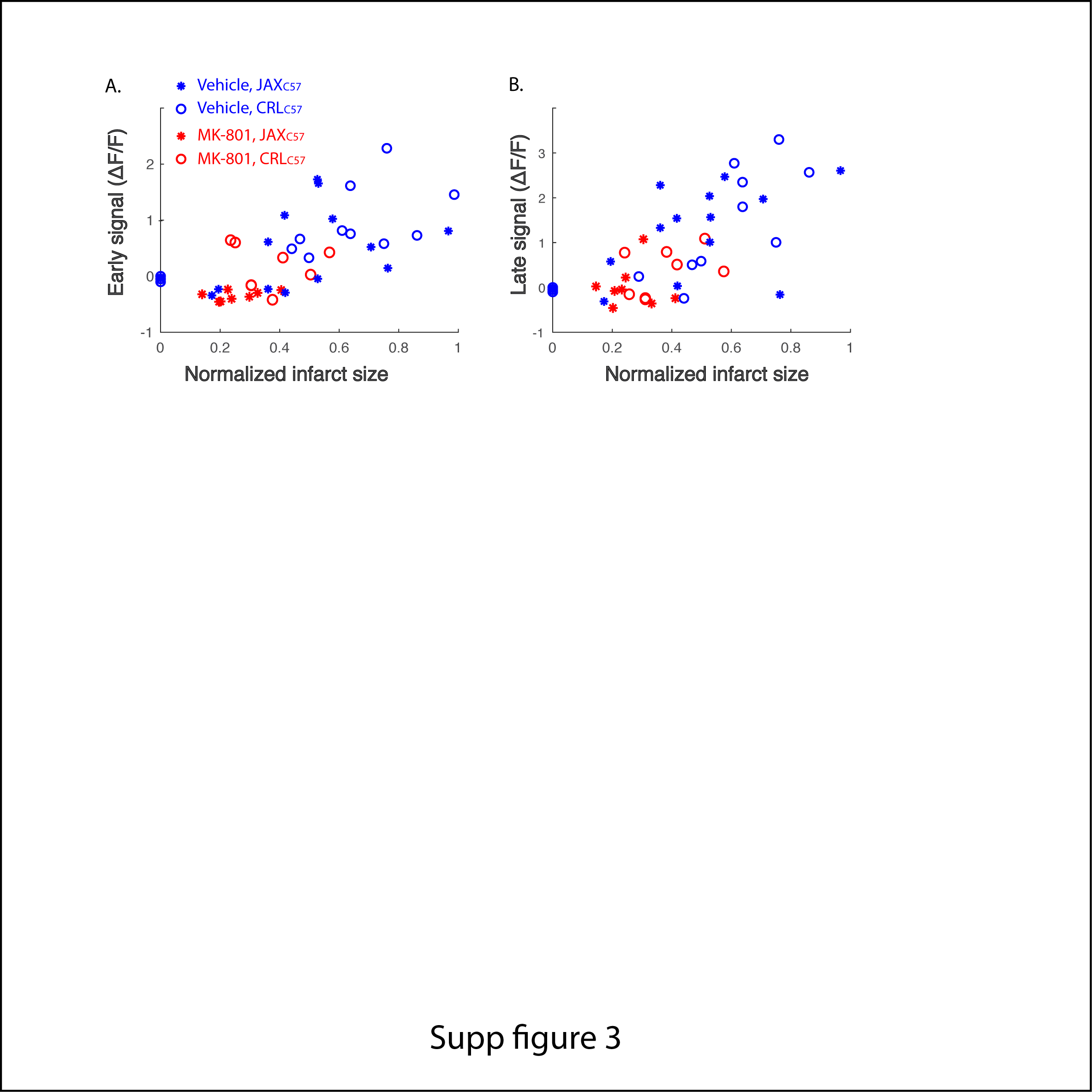

Supplement: Supplementary Figure 3 — Treatment with MK-801 significantly attenuates both the calcium signal and infarct volume following dMCAO. (A,B) Responses to pretreatment of MK-801 result both in a decrease in infarct size and mean early (A) and late (B) signal. JaxC57Asterisks; CRLC57 circles. JaxC57Asterisks; CRLC57 circles. [file Image_3.TIF]
